# High Risk for Tuberculosis in Hospital Physicians, Peru

**DOI:** 10.3201/eid0807.010506

**Published:** 2002-07

**Authors:** Nilo Bonifacio, Mayuko Saito, Robert H Gilman, Fay Leung, Nancy Cordova Chavez, Jesús Chacaltana Huarcaya, Carlos Vera Quispe

**Affiliations:** * Hospital Nacional Daniel A. Carrion, Lima, Peru; † Asociacion Benefica Proyectos en Informática, Salud, Medicina y Agricultura (Prisma), Lima, Peru; ‡ Johns Hopkins University Bloomberg School of Public Health, Baltimore, MD, USA; § Universidad Peruana Cayetano Heredia, Lima, Peru

**To the Editor:** Occupational exposure to *Mycobacterium tuberculosis* poses a major risk to medical staff worldwide. In areas of high tuberculosis (TB) incidence such as Peru (228–364 cases/100,000 [1,2]), the risk for hospital workers may be 40 times greater than that for the general population (3). Prospective studies to evaluate the precise occupational risk to medical staff in developing countries with a high incidence of TB disease are lacking. We evaluated the incidence of TB skin test (TST) conversion and TB disease in interns and residents in a teaching hospital in Lima, Peru.

Our study included 54 medical interns and 45 residents who began training in April 2000 at the Carrión Hospital, where all patients with TB are housed in wards without respiratory isolation. Each study participant had an initial evaluation before beginning hospital training. TST and chest radiographs were performed on entry into the study and 11 months later. TSTs were performed by the standard Mantoux technique, that is, intradermal injection of purified protein derivative (5 tuberculin units/0.1 mL) (Connaught Ltd., Ontario, Canada). Indurations were measured 48–72 hours later. A positive result was defined as an induration >10 mm. TST conversion from a negative to a positive result was defined as an increase of >10 mm in induration, according to criteria of the Centers for Disease Control and Prevention (CDC) (4). Every 3 months, physicians were screened for signs and symptoms of TB, and at 9 months they were interviewed for risk factors such as area of service, contact with active TB cases, and useage of an approved mask (N95 type). All statistical analyses were performed by using STATA 7.0 (Stata Corporation, College Station, TX).

Ninety-eight interns and residents (mean age 29.2 years ± 4.1 SD) were evaluated with an initial TST. One resident declined participation. Fifty-nine percent of the participants were TST positive at the initial evaluation. The presence of one BCG scar was not correlated with initially positive TST results. However, positivity in participants with two or more BCG scars was significantly higher (odds ratio 8.6; 95% confidence interval 1.8 to 79.5; p=0.002). Approximately two thirds (66/97) of participants recalled contact with an active TB case before the study period. All eight physicians whose relatives had been treated for TB were initially TST positive (p=0.01).

Of the 40 physicians who were negative at the initial TST, 35 (88%) were tested again 11 months later. Five residents did not have a TST. In one of these, an intensive-care resident, active pleural TB developed; the other residents remained well after 1 year. Five of the 35 physicians retested after 1 year had converted; 1 of these 5 also had pleural TB. Thus, 2 (5%) of 40 initially TST-negative physicians had acquired active TB, for an annual incidence of 2% (2/98).

The annual TST conversion rate for TST-negative physicians was 17% (6/36, including the TB patient without a follow-up TST). In addition, 11 (31%) of 35 persons with an initial negative TST result were positive (>10 mm) on second testing, but the increase in induration was <10 mm. No significant differences were observed between conversion rates in interns and residents.

Ninety-five physicians responded to the questionnaire on mask use. Over 9 months, 87 (92%) of 95 trainees had treated patients with active TB (mean number of cases 14 ± 14 SD). In the TST-negative group who were retested, physicians who converted had significantly more contact with patients known to have active TB than physicians who did not convert (24 ± 11 SD, respectively, vs. 8 ± 7 SD, p=0.003). During this period of follow-up, no physician was aware of his having been exposed to a TB patient in the physician’s home. No other risk factors (age, gender, area of service, participation in intubation or autopsy, approved mask usage, number of BCG scars) differed significantly between converters and nonconverters.

No physician reported consistently using a mask when examining patients with suspected TB or respiratory symptoms. Only 7 (7.4%) of 95 physicians reported that they consistently used a mask when examining active TB cases. Furthermore, 51 (54%) physicians never used a mask when examining a patient. Of the six physicians who converted, two reported never having used a mask, two reported mask use when working with diagnosed TB patients, and two reported sometimes using masks when working with TB patients.

Physicians exposed to a large number of TB cases at a public hospital had a 17% annual TST conversion rate. This rate is much higher than the 3% conversion rate in people living in a poor, overcrowded urban setting (2). Studies in industrialized countries show annual conversion rates ranging from 0.1% to 2% in unexposed employees and 1% to 10% in highly exposed health-care workers (5).

Our study also demonstrated a high incidence of symptomatic TB in Peruvian physicians. The 2% rate reported in this study is 10–15 times higher than that reported for the general population (6) and is similar to that in nurses caring for advanced TB patients in England during the 1930s (7).

The high TST conversion rate in physicians is most likely due to exposure to TB in the hospital. Boosting may at times produce large reactions and in all serial TB studies will be a potential confounder (8). To decrease the likelihood that boosting had occurred, we used stringent CDC criteria for conversion. The high incidence of active TB in the physicians strongly suggests that most conversions were due to TB transmission rather than boosting.

Additionally, the high conversion rate (two of five) in those who reported consistent mask use when caring for active TB cases may suggest overreporting of mask use, poor adjustment of the mask, contact with unsuspected active cases (9), or contact with a contaminated environment.

This high TST conversion rate and incidence of TB demonstrate the inadequacy of hospital infection control measures. In Peru, both unsuspected active TB and multidrug-resistant TB are highly prevalent (9). Rapid detection and respiratory isolation of patients with active or suspected TB are rarely practiced.

In conclusion, Peruvian physicians have an extremely high risk of TST conversion and active TB. Hospitals in developing countries need to design and implement effective and appropriate infection control measures such as appropriate mask usage, sputum testing, and rapid reporting of MTB smears of all patients with respiratory symptoms, as well as respiratory control for smear-positive TB cases (10).
